# Reactivity and Hydration Property of Synthetic Air Quenched Slag with Different Chemical Compositions

**DOI:** 10.3390/ma12060932

**Published:** 2019-03-20

**Authors:** Hui Wang, Yali Wang, Suping Cui, Jianfeng Wang

**Affiliations:** College of Materials Science and Engineering, Key Laboratory of Advanced Functional Materials, Education Ministry of China, Beijing University of Technology, Beijing 100124, China; wanghui606@outlook.com (H.W.); wangyali1978@bjut.edu.cn (Y.W.); wangjianfeng@bjut.edu.cn (J.W.)

**Keywords:** air quenched slag, chemical composition ratios, amorphous content, compressive strength, hydration

## Abstract

Air quenched slag is processed by a fast air cooling method which is developed with the advantages of recovering heat from molten slag and water conservation compared to the water quenching method. Air quenched slags with different chemical compositions are synthesized in the lab by designing three chemical composition ratios: (CaO + MgO)/(SiO_2_ + Al_2_O_3_), CaO/MgO and SiO_2_/Al_2_O_3_, which are donated as CM/SA, C/M and S/A, respectively. The effect of different chemical compositions on the phase compositions of synthetic air quenched slag, the strength and hydration properties of slag blends were investigated by using various characterization techniques. The results show that the amorphous content of air quenched slag decreased with the increasing basicity CM/SA of slag. The S/A ratio of slag was the dominant factor for the compressive strength of slag blends at 28 days and negatively correlated with strength. Decreasing the S/A ratio of slag increased the reactivity of slag and its reaction degree indicated by higher hydration heat release, lower CH content, greater chemical combined water amount and denser microstructure. Moreover, thermodynamic modelling revealed that a higher S/A of slag leads to the increase of C-(A)-S-H and AFt contents, whilst decreasing the amounts of Ht, AFm-SO_4_ phases and the total volume of hydrates.

## 1. Introduction

Blast furnace slag is a by-product of iron making with a generation rate of about 300 kg for one ton of iron [1]. The molten slag, which is produced by earthy constituents of iron ore, limestone flux and coke ash at 1350–1550 °C, is mainly consists of silica, alumina, lime and magnesia [2,3,4,5]. The molten slag are usually cooled rapidly by water quenching method to form granular solid slag with amorphous structure [2,3,5]. When finely ground, activated by chemical activators or thermal methods, this water quenched slag can exhibit excellent cementitious properties [3,6,7,8]. Thus, it has been widely used as supplementary cementitious material (SCM) in cement and concrete industry, which brings great economic and environmental benefits [9,10,11]. It is reported that approximately 800 kg of CO_2_ can be reduced if one ton of slag replaces one ton of clinker [12]. However, the water quenching method exposes some drawbacks when cooling the molten slag. Besides the consumption of water and additional energy to dry the obtained wet slag, the main disadvantage of water quenching method is the wasting of huge sensible heat energy of molten slag which is about 1.77 GJ/ton [13,14,15].

For the main purpose of recovering the heat energy from molten slag, alternative dry cooling technologies have received considerable attention [14,15,16]. In dry cooling process, the molten slag is almost all crushed and granulated by mechanical force and the generated droplets are quenched fast using high-speed air, with simultaneous heat recovery. Compared to water quenching, dry cooling method not only offers the basis for heat recovery from molten slag but also has the advantages of water conservation and no drying costs for slag utilization. However, up to now except for a few reported semi-industrial scale applications, most of dry slag granulation trials are still in lab-scale [15,17,18]. These researches were mainly focused on the equipment optimization of dry granulation system, the particle size and amorphous content of resulting slag droplets [15,16,17] but the reactivity and hydration properties of slag granulated by dry cooling process are not well investigated. Liu et al. [19] reported a 28-day compressive strength of 49.5 MPa for a blended cement with dry granulation slag. A later study found that increasing the replacement level of this dry granulation slag from 33.33% to 60%, the 28-day compressive strength of slag cement decreased first, then increased [20].

In the above works, only one type of slag with a specific chemical composition was investigated. However, slag chemical composition changes depending on the regions, the source of ores and the blast furnace operations. It was reported that the reactivity of slag is highly dependent on its chemical composition; slag with different chemical compositions shows different mineralogical composition, reactivity and hydration performance [2,5,9,21,22,23,24,25,26]. Several empirical moduli (e.g., (CaO + MgO + Al_2_O_3_)/SiO_2_)) based on slag main oxides CaO-MgO-SiO_2_-Al_2_O_3_ have been summarized to assess and predict the reactivity or mechanical property of slag but a number of studies showed that the proposed moduli could not give a valid and reliable estimation of slag strength performances [2,5,21,25]. Moreover, Mostafa et al. [24] indicated that the effect of cooling conditions on the amorphous content and the structure of slag is also governed by its chemical composition. Therefore, with the growing interest in using dry processed slag as SCM, an understanding of the correlation between the chemical compositions, reactivity and hydration property of obtained slag is needed, which is also helpful to further develop the dry cooling method and optimize processing parameters.

In this work, the slag with different chemical compositions were synthesized by a simple lab air quenching method, which is called air quenched slag. The chemical compositions of air quenched slag were designed by the uniform design method indicated as three ratios: the basicity (CaO + MgO)/(SiO_2_ + Al_2_O_3_), the ratio of alkaline oxide CaO/MgO and the ratio of acid oxide SiO_2_/Al_2_O_3_. The aim of the present study is to investigate the reactivity and hydration properties of air quenched slag with different chemical compositions. The compressive strength results were correlated to the chemical composition ratios of air quenched slag. The hydration kinetics, hydrates and microstructure of air quenched slag cement were analyzed using various characterization techniques including calorimetry, X-ray diffraction (XRD), thermogravimetry-differential scanning calorimetry (TG-DSC), scanning electron microscopy (SEM) and nitrogen adsorption method. Additionally, thermodynamic modelling was carried out to model the hydrate phase assemblages and estimate the reaction degree of slag.

## 2. Materials and Methods

### 2.1. Synthetic Air Quenched Slags

#### 2.1.1. Raw Materials

Considering the dry processed slag is unavailable from factories, a better way out to get this slag with different chemical compositions is to synthesize slag. In order to get similar chemical composition with the practical slag, limestone and industrial fly ash were used as alternative materials for limestone flux and coke ash which used in practical slag making process. The limestone mainly provides CaO for the synthetic slag and the fly ash supplies SiO_2_ and Al_2_O_3_ due to the similar constituents with coke ash. Besides, pure chemical reagents MgO and SiO_2_ were also used to compensate the needed contents of these oxides for synthetic slags. The chemical compositions of limestone and fly ash determined by X-ray fluorescence (XRF) are shown in Table 1. By using these industrial materials, some minor oxides such as FeO, SO_3_ and K_2_O can be introduced into slag, which makes the synthetic slag closer to the practical slag.

#### 2.1.2. Design of Slag Chemical Compositions

For typical blast furnace slag, the composition ranges of main oxides (wt.%) are CaO (30–50%), SiO_2_ (27–40%), MgO (1–10%), Al_2_O_3_ (5–15%) [3], which make up more than 95% content of slag. In our study, three ratios from CaO, SiO_2_, Al_2_O_3_ and MgO were proposed to design the chemical compositions of synthetic slag, which were indicated as the basicity [CM/SA = (CaO + MgO)/(SiO_2_ + Al_2_O_3_)], the ratio of alkaline oxides (C/M = CaO/MgO) and the ratio of acid oxides (S/A = SiO_2_/Al_2_O_3_). By using uniform design method [27,28], each ratio with ten levels were designed based on the above composition ranges of the four oxides. The U10(10^10^) uniform design table [28] was used and the designed chemical compositions of ten batch slags are listed in Table 2.

Then, the proportions of four raw materials (limestone, fly ash, SiO_2_ reagent and MgO reagent) were calculated by setting up and solving simultaneous equations on the basis of the designed three chemical composition ratios (Table 2), which is exactly similar to the proportioning method of cement raw meal using the lime saturation factor, silica ratio and alumina ratio [2]. This method can figure out the proportions of each raw material before calcination by excel programming solver. The calculated results are shown in Table 3.

#### 2.1.3. Melting and Air Quenching Process

**Figure 1:**
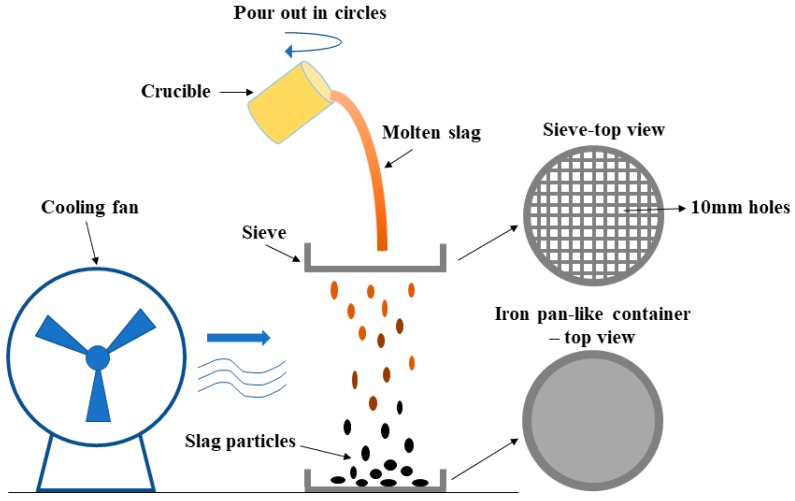
Schematic diagram of lab-scale simple air quenching process.

In this experiment, ten samples were obtained by mixing powders of limestone, fly ash, SiO_2_ reagent and MgO reagent in accordance with Table 3. The samples in corundum crucibles were heated in the furnace from room temperature to 950 °C at the rate of 10 °C/min and kept for half an hour at 950 °C for the fully decomposition of limestone powder, then continually heated to 1450 °C at the same rate and kept for half an hour at 1450 °C to melt the sample and homogenize the chemical compositions. Afterwards, the liquid molten slag was taken out of furnace, immediately poured out in circles manually passing through a disc iron sieve with 10 mm bore diameter to granulate the slag. At the same time, the molten slag was air quenched by a fan to obtain glassy structure. The solid slags are collected by an iron pan-like container. The schematic diagram of lab-scale air quenching process is shown in Figure 1.

### 2.2. Mortar and Paste Preparation

**Figure 2:**
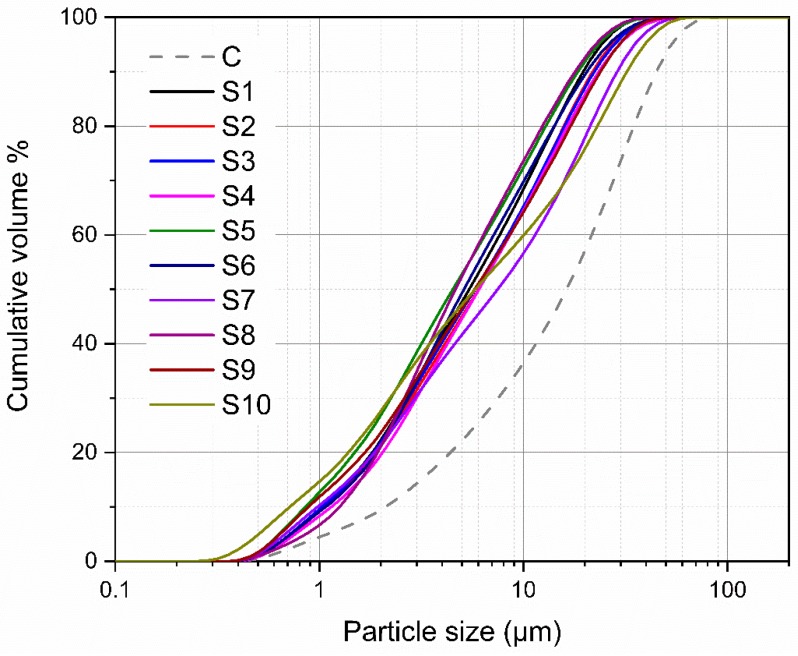
Particle size distribution of air quenched slags and cement.

To study the hydration property of synthetic slag in cement system, ten air quenched slags were blended with cement at a slag/cement ratio of 1:1 to produce cementitious binders, which were designated as CS1, CS2, CS3, ..., CS9, CS10, respectively. The neat cement binder (designated as C) was also prepared and used as a reference. All slags were ground in a ball mill to achieve similar fineness and the particle size distribution (PSD) and Blaine specific surface area of air quenched slag powders are shown in Figure 2 and Table 4. The cement used in the experiment was Portland cement (P.I 42.5R). The chemical and mineralogical compositions of cement (wt.%) were as follows: SiO_2_-21.65, Al_2_O_3_-4.05, Fe_2_O_3_-2.73, CaO-64.14, MgO-1.81, SO_3_-2.64, Na_2_Oeq-0.66, f-CaO-0.60, LOI-1.71, C_3_S-58.78, C_2_S-21.38, C_3_A-6.49, C_4_AF-8.77 (C = CaO, S = SiO_2_, A = Al_2_O_3_, F = Fe_2_O_3_). The PSD and Blaine specific surface area of cement is also given in Figure 2 and Table 4. 

Mortar specimens in size of 40 × 40 × 160 mm^3^ were prepared with a water/binder ratio of 0.50 and binder/sand ratio of 1:3. During the first 24 h, the mortars were cured in a moisture room with 95% relative humidity at 20 °C. Then, mortar samples were demolded and cured in water at 20 °C up to 7 and 28 days for strength test. The compressive strength tests were performed according to Chinese standard GB/T 17671-1999 [29].

Paste samples of cementitious binder with a water/binder ratio of 0.50 were cast in cubic mold in size of 20 × 20 × 20 mm^3^, six cubes for each formulation. All pastes were cured in the same conditions with mortar bars up to 3, 7 and 28 days.

### 2.3. Characterization Methods

For air quenched slags, the chemical compositions were analyzed by XRF on a Shimadzu 1800x instrument (Kyoto, Japan). X-ray diffraction (XRD) was conducted on a Shimadzu XRD-7000 diffractometer with a Cu Kα anode operating at 40 kV and 30 mA. The scanning was performed in a 2θ range of 10–70° with a step size of 0.02°. Rietveld analysis was used for the quantitative phase analysis. The amorphous phase content of slag was determined with a second XRD-run using α-Al_2_O_3_ as an internal standard. The contents of phases in slag were calculated by knowing the specific weight percent of the added standard to the slag in each XRD run.

Isothermal calorimetry was performed using a thermal activity monitor (TAM) instrument with an eight-channel heat flow calorimeter, at a temperature of 30 °C. About 4.5 g of paste with the same mix proportion as described in Section 2.2 was mixed externally in an admix ampoule and loaded in the TAM calorimeter to measure the heat release during the first 3 days.

Paste samples were investigated by XRD, thermogravimetric analysis (TGA), scanning electron microscopy (SEM) and Nitrogen adsorption test after 3, 7 and 28 days of hydration. For XRD and TGA analysis, the hardened paste samples were immersed in ethyl alcohol to stop hydration. Before measurement, the samples were dried at 40 °C for 24 h, then ground to powder with fineness below 63 μm. XRD was performed as described above for anhydrous slag but without Rietveld analysis. TGA was carried out using a Netzsch STA449C thermal analyzer (Selb, Germany) between 35 and 1000 °C at a heating rate of 20 K/min under nitrogen atmosphere. The combined water content *w*(*t*) and the Ca(OH)_2_ content *m_CH_*(*t*) were determined using the weight loss Δ*m*(*t*) of paste sample at the temperature intervals 35–550 °C and 400–500 °C (Equation (1)) respectively [23,30].

(1) $$m_{CH}\left(t\right)=\Delta m_{400-500^{{}^{\circ}}C}\left(t\right)\cdot \frac{M_{Ca\left(OH\right)2}}{M_{H2O}}$$

where *M_Ca_*_(*OH*)2_ and *M_H_*_2*O*_ are the Ca(OH)_2_ and water molar mass (g/mol), respectively.

After dried at 40 °C for 24 h, some broken paste samples were coated with gold film. The morphology of fracture surface of hydrated paste was analyzed using a FEI Quanta FEG 650 SEM (Hillsboro, OR, USA) equipped with energy dispersive spectroscopy (EDS) at an accelerating voltage of 20 kV. Nitrogen adsorption was performed on a Micromeritics TriStar II 3020 Surface Area and Pore Size Analyzer (Norcross, GA, USA). About 0.2 g of paste sample was used for each analysis with vacuum drying and degassing before the experiment.

Phase assemblages were modelled using Gibbs free energy minimization program GEM-Selektor v.3.3.5 (GEMS) [31,32,33]. Thermodynamic data was taken from the PSI-GEMS database [34] and cement specific database (Cemdata18.01) [35,36]. The amorphous phase of slag was assumed to dissolve uniformly. The C-S-H phase is modelled with a constant Al/Si of 0.14 based on the EDS analysis of similar slag cement paste in other studies [37].

## 3. Results and Discussion

### 3.1. Characterization of Synthetic Air Quenched Slags

#### 3.1.1. Chemical Compositions of Slags

Table 5 shows the chemical composition of synthetic air quenched slag determined by XRF. In Table 5, the three chemical composition ratios CM/SA, C/M and S/A of obtained slags are changed regularly following the expected trends of the original design (Table 2). The values of three ratios are globally comparable with the designed ones, even though a small composition deviation exist due to the preparation and melting processes.

**Figure 3:**
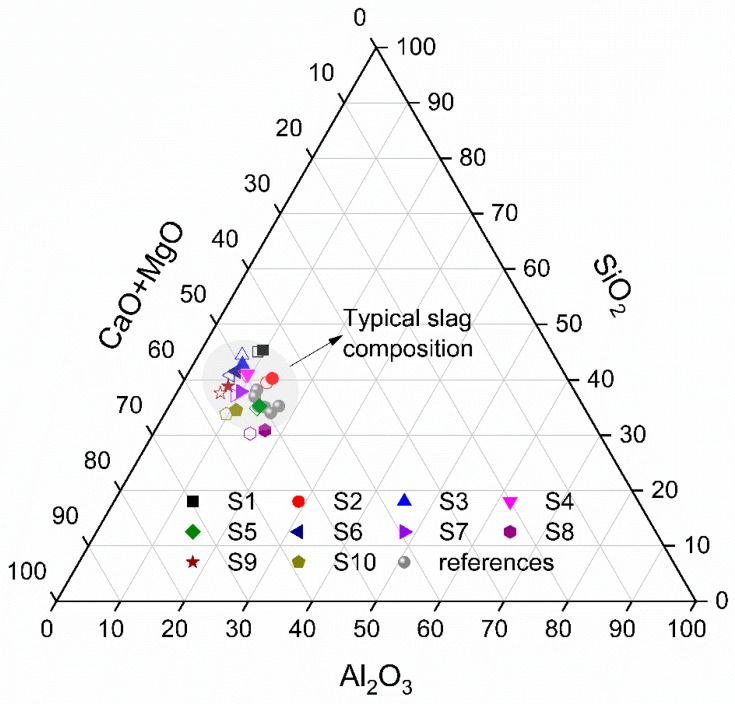
Chemical compositions of air quenched slags in the (CaO + MgO)-SiO_2_-Al_2_O_3_ ternary system (in wt.%). The solid points are taken from XRF results of synthetic slags. The hollow points which have the same shape with the solid points are the designed compositions of slags in Table 2. The points marked as references are the XRF compositions of several water quenched slags from references [2,3,5].

To compare the main oxides of slags further, the chemical compositions of synthetic slags in the ternary system of (CaO + MgO)-SiO_2_-Al_2_O_3_ are depicted in Figure 3. This depiction of ternary system for slag composition only considers the indicated four main oxides in Table 5 normalized to 100 wt.%. In Figure 3, all synthetic slags show similar oxide compositions with the designed slags. The differences between theoretical designed value and XRF result are in the range of 0–1.68 wt.%. In addition, the composition positions of obtained air quenched slags are almost in the typical composition region of blast furnace slag and close to those of water quenched slags reported in other references [2,3,5]. This confirms the normal chemical composition of the synthetic slags and the feasibility of the preparation method.

#### 3.1.2. Appearance of Slags

**Figure 4:**
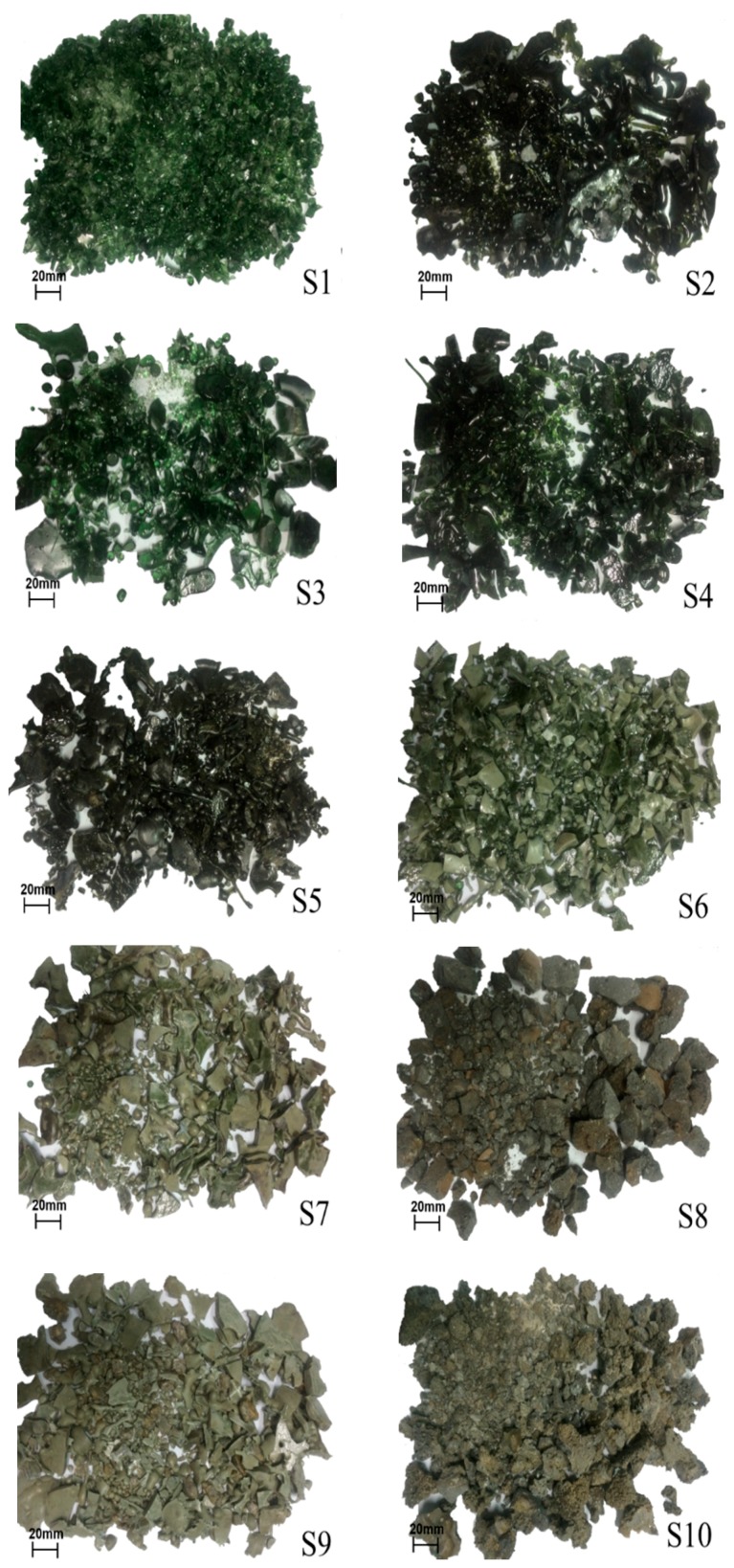
Photographs of synthetic air quenched slags.

Figure 4 shows the photographs of synthetic air quenched slags. Overall, all air quenched slags were in the shape of irregular bulks and spherical grains with different sizes due to the simple air quenching process. The dark green color of slags is related to the ferrous iron in raw materials, which is observed in the blast furnace slag. The surface of air quenched slag is smooth, and the structure is dense with low porosity. As shown in Figure 4, the slags S1–S4 with the basicity CM/SA < 1.0 showed considerable transparent and glassy materials, while some opaque crystalline materials with an uneven distribution were formed in S5 and S6 whose basicity are around 1.0–1.05. For slags of S7–S10 with the basicity CM/SA > 1.05, only minor amorphous phase is visible and the slags are almost fully crystallized in a stone-like structure. During the cooling process, S8 and S10 showed bad fluidity and fast crystallization after taking out of furnace, which is due to the effect of high basicity for these slags. Based on above observations, it could be concluded that with the increase of CM/SA, the air quenched slags became opaquer and contained less amorphous phase.

#### 3.1.3. Phase Compositions and Amorphous Content

**Figure 5:**
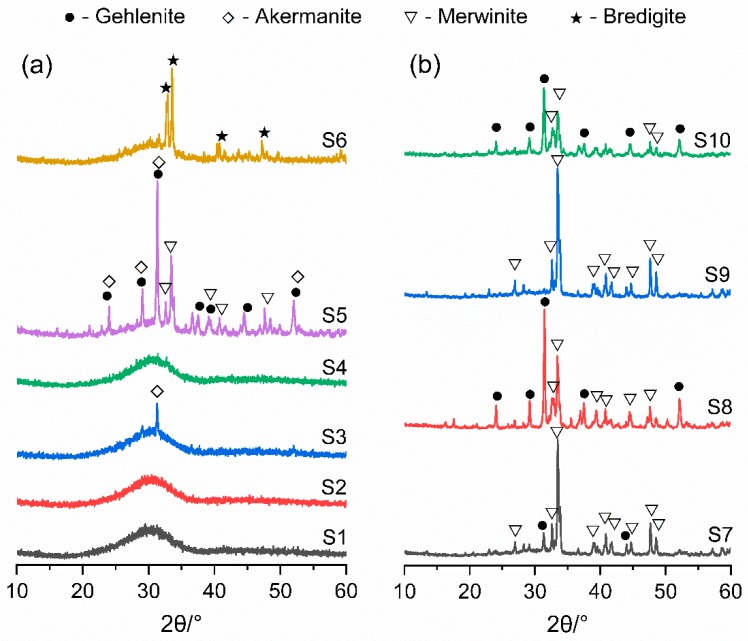
X-ray diffraction (XRD) patterns of anhydrous slags (**a**) CM/SA ≤ 1.05 (**b**) CM/SA > 1.05.

Figure 5 shows the XRD patterns of air quenched slags. In Figure 5a, an amorphous hump centered at 22–38° with almost invisible crystalline peaks is observed for S1–S4. The small diffraction peak in S3 shows minor content of crystalline phase akermanite. However, in the case of S5 and S6, the amorphous hump decreased obviously and there are many sharp diffraction peaks appearing, which reveals the considerable amount of crystalline phases. In Figure 5b, many peaks of crystals are observed for S7–S10 and the amorphous hump is not obvious. The main minerals in air quenched slags are gehlenite (Ca_2_Al(AlSiO_7_)), akermanite (Ca_2_Mg(Si_2_O_7_)) and merwinite (Ca_3_Mg(SiO_4_)_2_), while some bredigite (Ca_7_Mg(SiO_4_)_4_) was formed in S6.

The phases compositions of slags were quantified by Rietveld analysis and the results are given in Table 6. The content of amorphous phase in air quenched slag decreased with the increase of basicity CM/SA (Table 5), while there seems no detectable effect was observed for the content of amorphous phase with the changing of C/M and S/A of slag. This is due to the high content of alkaline oxides which leading to the faster and stronger crystallization rate of slag during the cooling process. However, the type and amounts of crystalline phases were associated with the Al_2_O_3_ and MgO contents in slags (Table 5). Higher Al_2_O_3_ in slag increased the gehlenite content and more MgO resulted in the greater formation of akermanite and merwinite.

### 3.2. Compressive strength of Slag Blended Mortar

**Figure 6:**
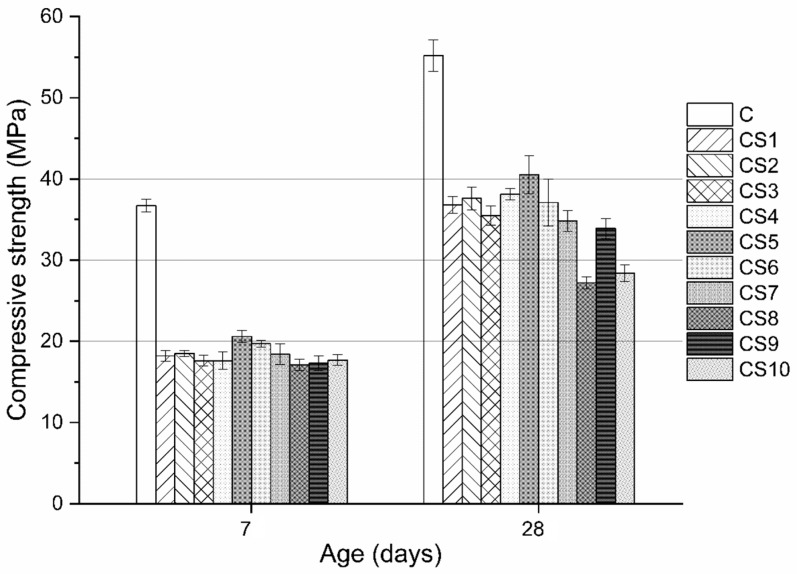
Compressive strength of slag blended mortars at 7 and 28 days.

Figure 6 shows the compressive strength of slag blended mortars at 7 and 28 days. The mortars blended with air quenched slags showed overall lower compressive strength compared to the neat cement at both ages which is due to the relatively low reactivity of air quenched slag than the cement. At 7 days, CS5 and CS6 obtained slightly higher strength than those of other slag mortars with comparable strength ~18 MPa. At 28 days, the strength of all slag mortars increased and CS5 obtained the highest strength ~40.5 MPa. Compared to CS5, the mortars CS1–CS4 and CS6 achieved slightly lower strength, while CS7–CS10 had much lower strength, especially CS8 and CS10 clearly showed the lowest strength with only 27–28 MPa at 28 days. This strength difference among slag mortars could be attributed to the different chemical and phase compositions of air quenched slags. S5 with 60.3% amorphous content showed the highest strength. However, S6 with higher amorphous content (73%) and S1–S4 containing almost all amorphous phase showed slightly lower strength than S5. This suggests that only the amorphous content is not an effective indicator to measure the reactivity of slag. Air quenched slag with low amorphous content ~60% could also have good strength which was in agreement with other literatures [9,24].

To further explore the relationship between the compressive strength of slag mortar, slag chemical composition ratios (Table 5) and amorphous content (Table 6), multiple regression analysis were employed, and the obtained equations are shown in Equations (2) and (3). In practice, a proper slag should be fully melted in furnace and show free fluidity when discharging to ensure the normal iron production and operation of blast furnace. But in the experiment the slags S8 and S10 were not fully melted and could not show free fluidity when taking out from the furnace, both slag could not satisfy the requirement for a proper slag. Thus, the data from CS8 and CS10 are not included in the regression analysis.

*Y*_7d_ = 29.60 − 7.019*X*_1_ + 0.198*X*_2_ − 0.085*X*_3_ − 0.064*X*_4_, *R* = 0.709 (2)

*Y*_28d_ = 38.26 + 0.857*X*_1_ + 0.222*X*_2_ − 1.073*X*_3_ + 0.011*X*_4_, *R* = 0.738 (3)

where *X*_1_ is CM/SA = (CaO + MgO)/(SiO_2_ + Al_2_O_3_), *X*_2_ is C/M = CaO/MgO, *X*_3_ is S/A = SiO_2_/Al_2_O_3_, *X*_4_ is the amorphous content of slag, *Y*_7d_ and *Y*_28d_ are the compressive strength of slag blended mortar at 7 and 28 days.

Equations (2) and (3) indicate that the chemical composition ratios of slag have more pronounced impact on the strength of slag mortar than amorphous content, as it is evident from the values of their corresponding coefficients in equations. For three chemical composition ratios, CM/SA was found to have a negative effect on 7-day strength while it showed a slight positive effect on strength at 28 days. C/M showed a positive but very weak correlation with strength at 7 and 28 days. Moreover, S/A was negatively correlated with strength at both ages and it became the most significant factor for strength at 28 days.

The obtained Equations (2) and (3) could roughly give the correlation of strength property with the slag chemical composition ratios and amorphous content but the fitting remains not satisfactory. This is because the strength of slag blends is a result of complicated combined effect of slag intrinsic property, hydration process and microstructure development, which could not consider them all in the relationships. A deeper investigation was therefore performed to better understand the interaction effect of hydration kinetics, hydration products and microstructure on the evolution of slag blended cement performance.

### 3.3. Hydration Properties of Slag Blended Paste

Considering the compressive strength results of slag blended mortars, the chemical and phase compositions of air quenched slag, hydration characterization of blended pastes with S1, S3 and S5 was carried out to further investigate the correlation between hydration property and slag chemical compositions. The reasons of choosing these slags are because they could approximately represent the acid slag (S1), neutral slag (S3) and basic slag (S5) respectively based on their chemical compositions. Additionally, S1 with 100% amorphous phase and S3 with 96.4% amorphous phase and minor crystalline phase, are close to the phase composition of practical slag. Slag S5 has only 60.3% amorphous content but showed the highest compressive strength. It is interesting to try to understand why it exhibited higher reactivity and strength property. Therefore, blended pastes with S1, S3 and S5 will be focused on for hydration investigation.

#### 3.3.1. Hydration Kinetics

**Figure 7:**
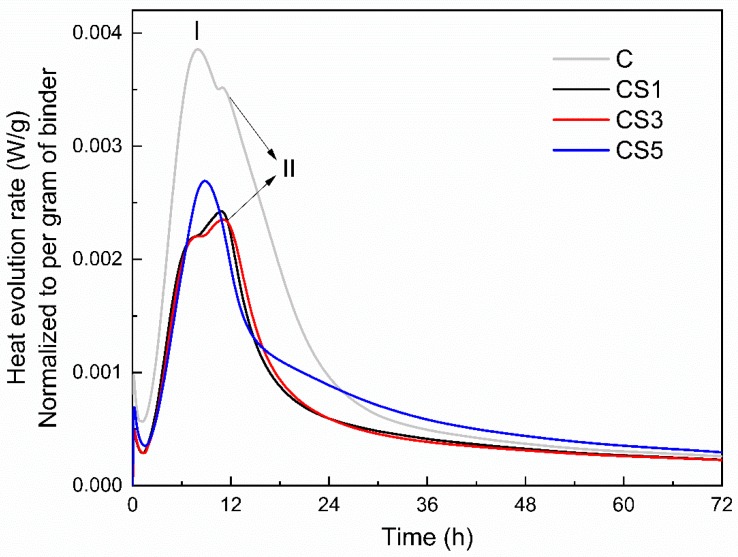
Heat evolution rate of slag blended pastes during 72 h of hydration.

The heat evolution rate of slag blended pastes is shown in Figure 7. The first peak I appears ~7.5 h was primarily caused by alite hydration [38,39,40]. Then, an additional slight peak II was observed for the neat cement, CS1 and CS3 after the deceleration of alite hydration at ~11 h, which is associated with the secondary aluminate reaction upon sulfate depletion [25].

The peak I corresponding to alite reaction for slag pastes is much lower than the neat cement, which is due to the 50% replacement of cement by air quenched slag. The addition of different slag mainly effect the aluminate reaction peak II. The occurrence and intensity of this peak were not constant in three slag blends: arising after 10 h for CS1 and CS3 but earlier and more enhanced for CS5. S5 seems to enhance the alite peak I as well but it is mainly due to the effect of an earlier sulfate depletion causing the overlap of alite peak with the aluminate peak for CS5 [25].

**Figure 8:**
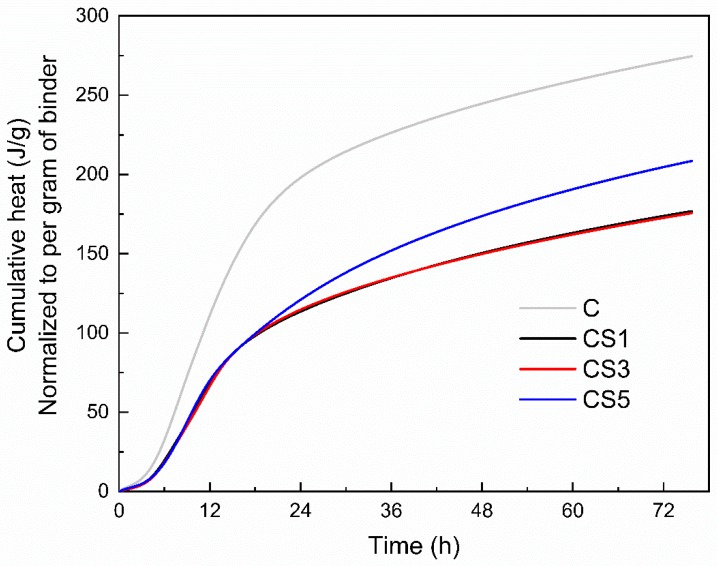
Cumulative heat of slag blended pastes during 72 h of hydration.

Figure 8 shows the cumulative heat of blended slag pastes at 72 h. The addition of air quenched slags evidently reduced the cumulative heat of blended pastes compared to that of neat cement. Higher heat evolution can be observed for CS5 than those of CS1 and CS3, while similar heats are released between CS1 and CS3. This indicates that S5 had higher reactivity than S1 and S3, which is consistent with the compressive strength results in Figure 6.

#### 3.3.2. Hydration Products

##### XRD Analysis

**Figure 9:**
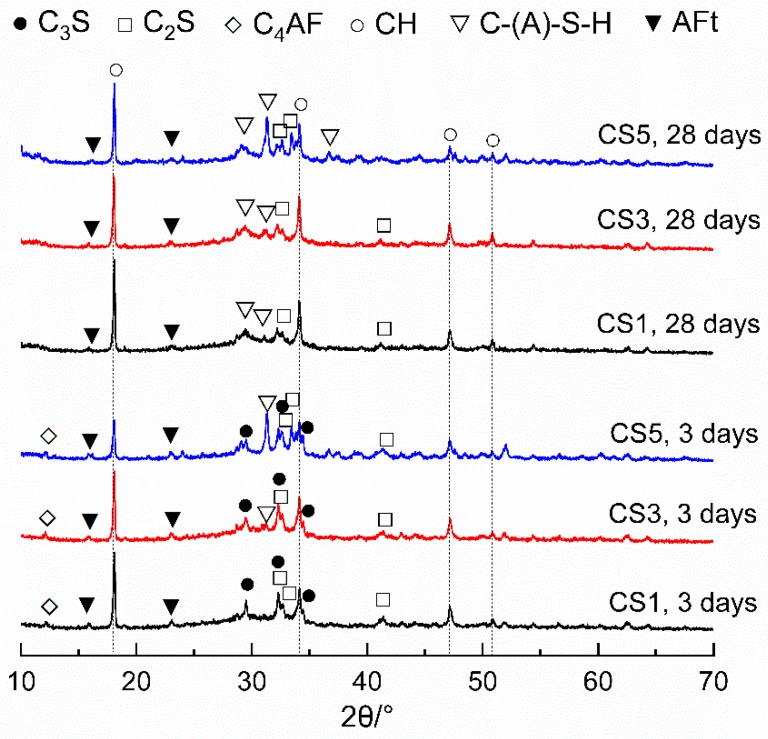
XRD patterns of slag pastes at 3 and 28 days. CH—portlandite, C-(A)-S-H—calcium silicate hydrate, AFt—ettringite.

**Figure 10:**
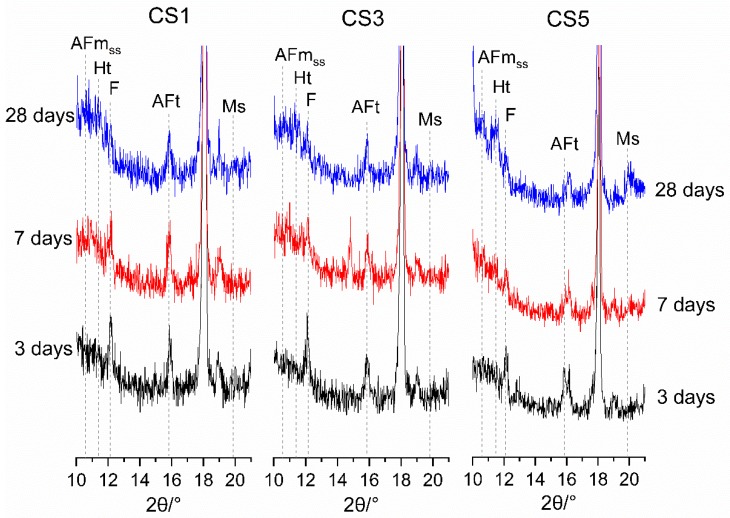
XRD patterns highlighting the phases from 10° to 21° for hydrated slag pastes. F—C_4_AF, Ht—hydrotalcite, AFt—ettringite, Ms—monosulfate, AFm_ss_—solid solution of CO_3_^2−^ and OH¯ substituted monosulfate.

XRD analysis of hydrated slag pastes (Figure 9 and Figure 10) reveals that the hydration products of paste blended with air quenched slag are portlandite (CH), calcium silicate hydrate (C-(A)-S-H), ettringite (AFt), hydrotalcite (Ht), monosulfate (Ms) and AFm_ss_. AFm_ss_ phase is a solid solution of CO_3_^2−^ and OH^−^ substituted monosulfate which had formed in some blended cements [41,42,43].

In Figure 9, the phases of C_3_S and C_2_S indicate the presence of residual cement. C_3_S presented at 3 days but disappeared at 28 days. Meanwhile, an increasing trend is observed for C-(A)-S-H and CH up to 28 days indicating the progressive hydration of slag pastes. At both ages, the CH peak for CS5 was slight lower than those of CS1 and CS3 which suggests the higher consumption of CH in CS5 due to the greater reaction of S5.

Figure 10 further highlights the effect of slag on the aluminous phases. A small amount of Ht was observed in all slag blends, confirming the hydration of air quenched slags. Ht peak appeared at 7 days and increased up to 28 days. At 28 days, CS5 showed a slightly higher amount of Ht than CS1 and CS3 since S5 had more MgO and Al_2_O_3_ which is in favor of the formation of Ht. The evolution of AFt, Ms and AFm_ss_ phases in slag pastes are also shown in Figure 10. As hydration proceeded, AFt was present obviously at 3 days and slightly increased to 7 days, then decreased at 28 days, while minor content of Ms and AFm_ss_ were appeared at 28 days. This is due to the transformation of AFt to Ms and AFm_ss_ after the sulfate depletion in blended system [25]. The effect of slag on the contents of AFt, Ms and AFm_ss_ is different depending on slag type. The AFt peak for CS5 was always lower than CS1 and CS3, while CS1 showed a highest AFt peak. At 28 days, the peaks of Ms and AFm_ss_ in CS5 were more pronounced than those of CS1 and CS3.

##### TG-DSC Analysis

**Figure 11:**
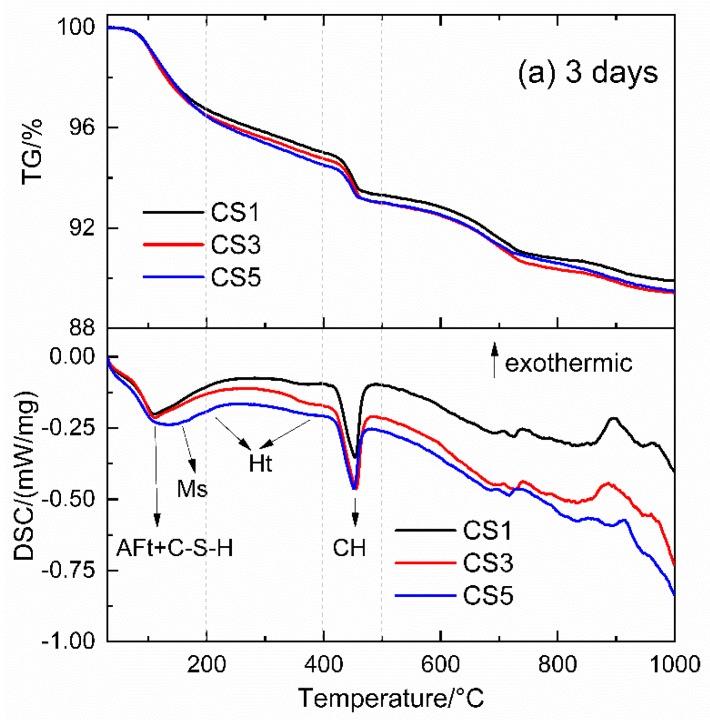

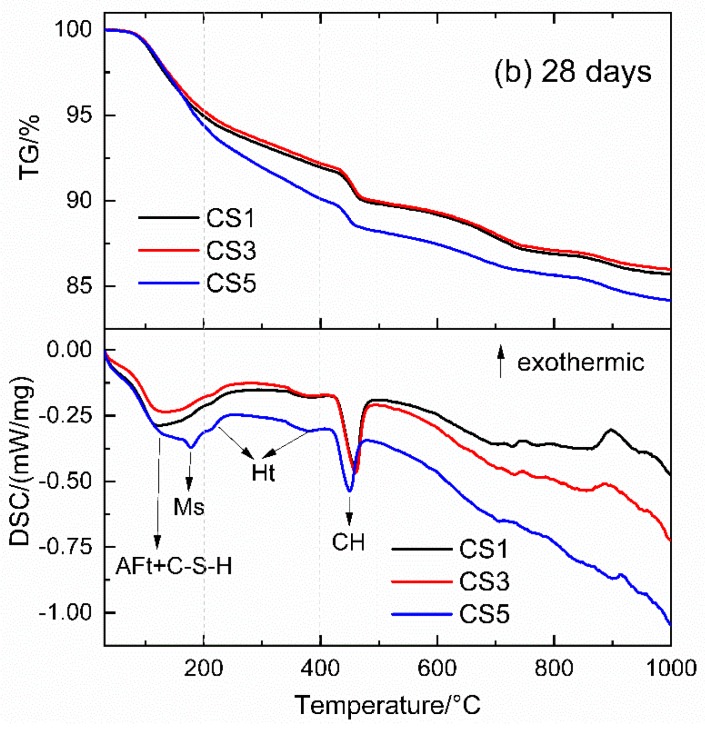
TG-DSC curves of slag pastes at (**a**) 3 days and (**b**) 28 days.

Figure 11 shows the TG-DSC curves of hydrated slag pastes at 3 and 28 days. The weight loss at 1000 °C for each slag paste is increasing due to continuous hydration and hydrates formation up to 28 days. The difference of weight losses at 1000 °C between pastes is small at 3 days but at 28 days it becomes larger for CS5 compared with CS1 and CS3.

In the DSC curves (Figure 11), three main endothermic peaks are observed for all slag pastes. One peak located below 200 °C was related to the dehydration of C-(A)-S-H, AFt and Ms, the next small peak between 200 and 400 °C was about the dehydration of Ht. The other peak between 400 and 500 °C was due to the decomposition of CH [44]. For CS5, the peak located below 200 °C was wider and shifted to higher temperature than CS1 and CS3 at both ages. The widening of this peak indicates that more hydrates such as C-(A)-S-H, AFt and Ms were formed in CS5, especially clear Ms peak appeared in CS5 at 28 days. In the temperature range between 200 and 400 °C, the Ht peak for all slag paste was almost invisible at 3 days but at 28 days it was enhanced and CS5 showed more intense Ht peak than those of CS1 and CS3. This is in agreement with the XRD result in Figure 10.

**Figure 12:**
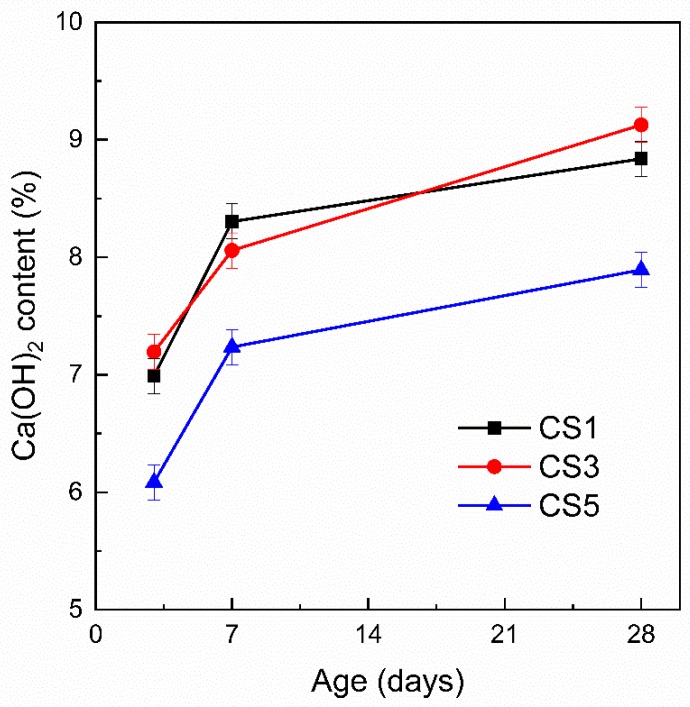
CH content of slag pastes at different ages determined by TGA.

The content of CH is usually used as an indicator of hydration degree of blended paste to estimate slag reactivity. A higher CH content means a lower consumption of CH, thus signifies the less reactivity of slag [45]. Figure 12 shows the CH content for slag pastes determined by TGA at different ages. CS5 gives the lowest content of CH at all ages than those of CS1 and CS3, while CS1 and CS3 show similar CH content with slight difference, which agrees with the CH peak evolution in XRD (Figure 9).

**Figure 13:**
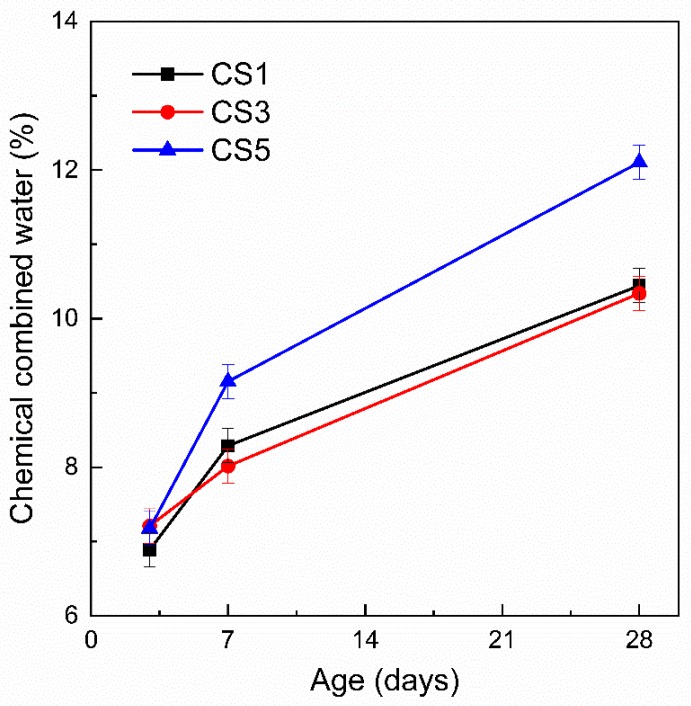
Chemical combined water of slag pastes at different ages determined by thermogravimetric analysis (TGA).

Moreover, the combined water content is also determined in Figure 13 to estimate slag reactivity [30]. Similar to the result of CH content, the combined water content evolution shows that there were more hydration products formed in CS5 after 3 days, indicating again the higher reactivity of S5 than S1 and S3.

#### 3.3.3. Microstructure of Slag Blended Paste

**Figure 14:**
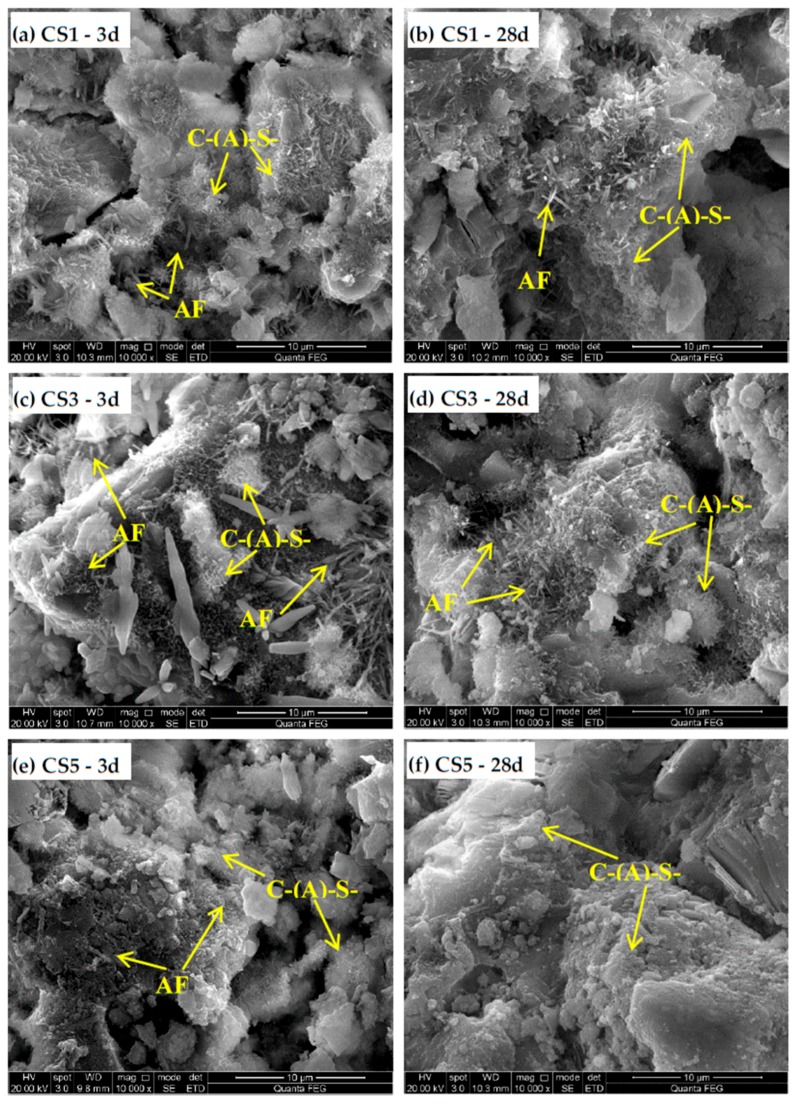
Scanning electron microscope (SEM) micrographs of hydrated slag pastes at 3 and 28 days. (**a**) CS1 at 3 days; (**b**)CS1 at 28 days; (**c**) CS3 at 3 days; (**d**) CS3 at 28 days; (**e**) CS5 at 3 days; (**f**) CS5 at 28 days.

Figure 14 shows the SEM micrographs of hydrated slag pastes at 3 and 28 days. At 3 days, early signs of C-(A)-S-H and rod-like AFt appearing on the surface of grains indicates significant hydration of slag pastes. C-(A)-S-H with honeycomb shape and fine needle shape in small clusters had an independent discrete distribution. In the micrographs of pastes cured for 28 days, a considerable amount of C-(A)-S-H with the structure of honeycomb and fine needle shape mixed with rod-like AFt are still existed in CS1 (Figure 14b) and CS3 (Figure 14d). However, C-(A)-S-H in case of CS5 (Figure 14f) showed a connected and centralized structure with less pores, the typical rod-like AFt was almost invisible and there was an intimate mixture of hydrated phases. This resulted in the more homogeneous and compact matrix of CS5 than CS1 and CS3 at 28 days.

**Figure 15:**
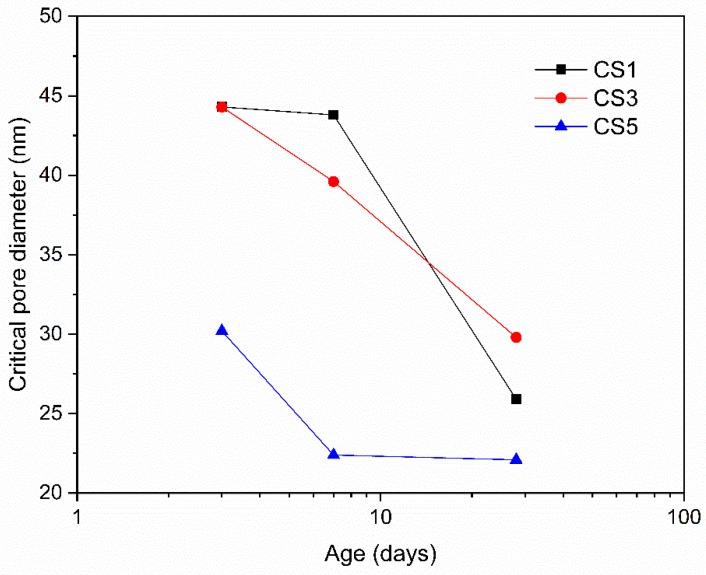
Critical pore size of hydrated slag pastes determined by nitrogen adsorption at 3, 7 and 28 days.

The critical pore size of hydrated slag pastes characterized by nitrogen adsorption method is shown in Figure 15. The critical pore size gradually decreased over time in all slag pastes. From 3 to 28 days, CS5 always showed lower critical pore size than CS1 and CS3, indicating a lower porosity and denser structure of CS5 matrix. This is in agreement with the denser microstructure with CS5 (Figure 14).

**Figure 16:**
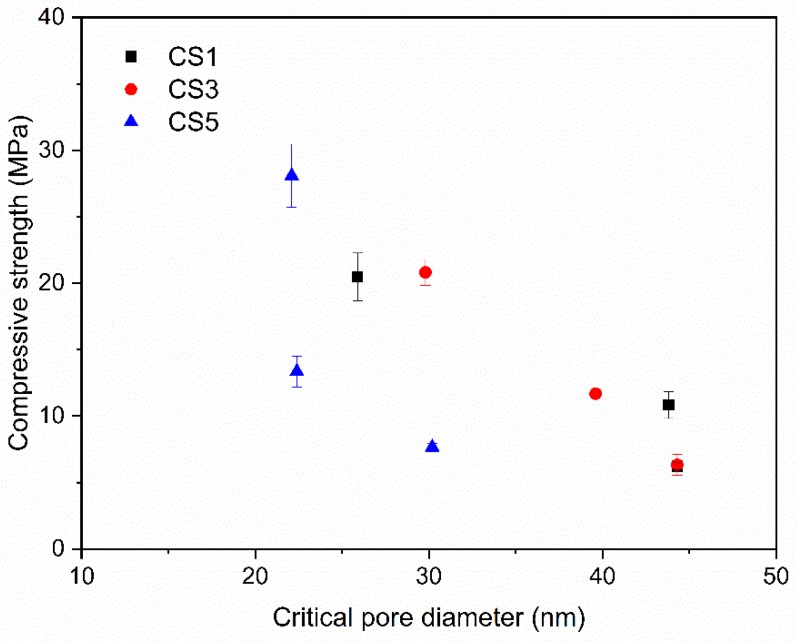
Relationship of compressive strength of slag blended pastes with critical pore size at 3, 7 and 28 days.

In Figure 16 a correlation between compressive strength and critical pore size was plotted. The compressive strength showed a negative correlation with the critical pore size. This suggests that the smaller critical pore size of CS5 indicating a lower total porosity contributed to its higher compressive strength at 28 days.

### 3.4. Discussion

Based on the aforementioned results, it is found that for three air quenched slags, S1 and S3 showed comparable reactivity while S5 exhibited higher reactivity than S1 and S3 as indicated by higher compressive strength, higher heat release, lower CH content, greater chemical combined water amount and denser microstructure of CS5, especially at 28 days. However, S5 had only 60.27% amorphous phase and considerably some crystalline phases compared to other two slags with almost all amorphous phase. How did S5 exert good reactivity with a low amorphous content? The higher reactivity of S5 is further confirmed by thermodynamic modelling, which showed that the degree of reaction (DoR) of S5 was higher than S1 and S3 at 28 days.

**Figure 17:**
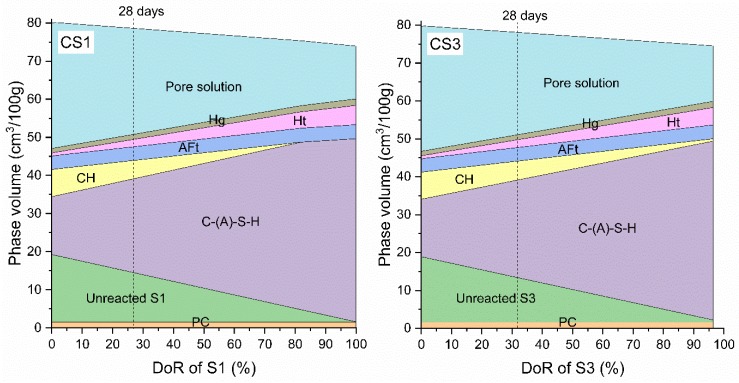

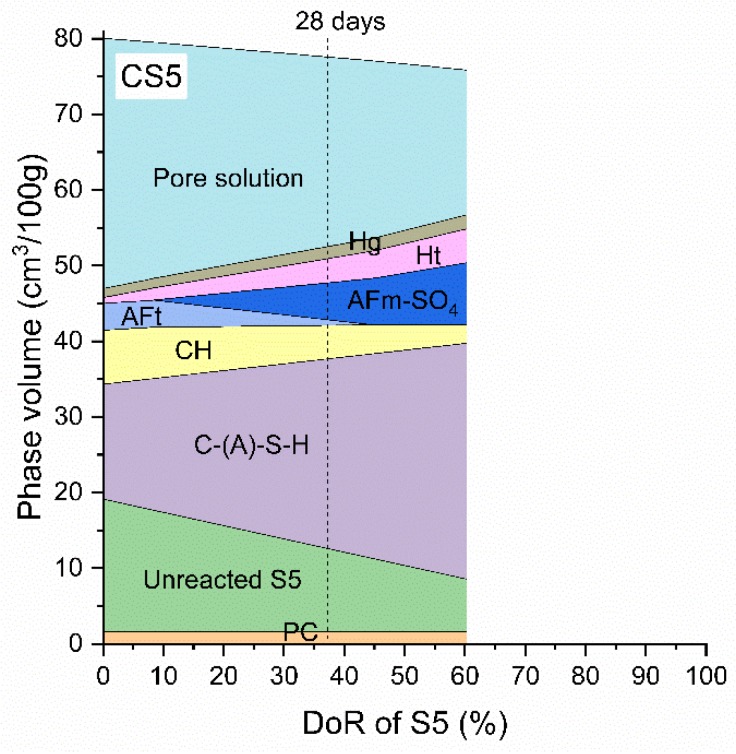
Modelled phase volume changes of slag blended pastes CS1, CS3 and CS5 as a function of DoR of slag by GEMS. Ht—hydrotalcite, Hg—iron bearing hydrogarnet, AFm-SO_4_—monosulfate, AFt—ettringite, CH—portlandite.

Figure 17 shows the modelled phase volume of three slag pastes changing with DoR of slag. The reaction degree of cement was assumed to be 90% which roughly corresponds to the actual situation of cement hydration after 28 days. Only the amorphous phase in air quenched slag was considered as reactive material and participated in hydration reaction. The chemical composition of amorphous phase in each slag was calculated by the difference of bulk chemical composition of slag (Table 5) and the corresponding contents of all crystal phases in slag (Table 6). Thus, the DoR of slag is shown up to 100% for S1, 96.4% for S3 and 60.3% for S5 in order to match with the maximum reacted content which is the amorphous content in each slag. The predicted hydrates in Figure 17 are C-(A)-S-H, CH, AFt, Ht and a small amount of Hg for three slag pastes. In CS5, AFm-SO_4_ is also predicated to form after ~10% reaction of S5. With the increase of reacted slag, portlandite gets consumed. The DoR of slag is estimated from the experimental CH content quantified by TGA (Figure 12). By picking up the modelled CH content which was equal to the CH content determined by TGA at 28 days, the corresponding DoR of slag at 28 days can be obtained and marked as the dashed lines in Figure 17. The obtained 28-day DoR for S1, S3 and S5 are 27.0%, 31.8% and 37.4%, respectively. The modelled phase contents of slag blended pastes corresponding to the obtained 28-day DoR of slag are shown in Table 7. It is observed that the modelled hydrates for three pastes at 28 days agree well both qualitatively and quantitatively with the experimental results discussed in XRD (Figure 9 and Figure 10) and TGA (Figure 11).

**Figure 18:**
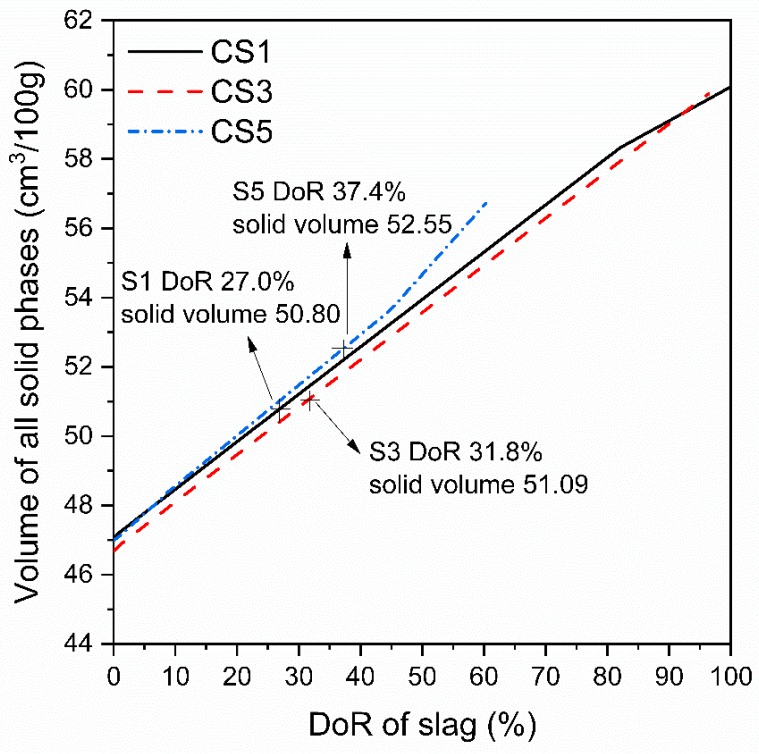
Relationship of solid phase volume for slag blended pastes with DoR of slag.

The volume of all solid phases in slag paste at 28 days was also determined based on the obtained DoR of slag as shown in Figure 18. When comparing three slag pastes, the higher volume of solid phases is clear visible for CS5 than those of CS1 and CS3 at 28 days. Although S5 had only 60.3% amorphous content but at 28 days the amorphous phase in S5 just reacted ~37.4%. However, this DoR of 37.4% for S5 is still higher than S1 and S3 at 28 days. Therefore, S5 with higher reaction degree and greater volume of solid phases resulted in the lower porosity of matrix and higher compressive strength of CS5. In the case of CS1 and CS3, S3 had higher DoR and slight greater solid phase volume than S1, thus one would expect a higher compressive strength of CS3. However, CS3 showed a little bit larger critical pore size than CS1 at 28 days which indicated a slightly higher porosity of CS3. This combined effect of hydrates and microstructure would lead to the comparable strength of both pastes at 28 days.

At a given DoR of slag less than 60% shown in Figure 18, CS5 always had a slightly higher volume of solid phases while S3 showed the lowest one. Figure 17 shows that the type and amount of hydrates formed in blended system vary significantly with different slags. This difference is mainly dependent on the chemical compositions of slag. It seems that air quenched slag with a low S/A ratio or rich in Al_2_O_3_ content could have higher reactivity and more solid phase volume. Moreover, the experimental hydration of slag blended pastes was also found to be strongly influenced by slag composition, most likely S/A of slag. The blended paste containing slag with a lower S/A ratio was observed to show more hydration heat release, higher CH consumption, greater chemical combined water amount and denser structure. However, no clear effect was observed for the hydration of slag paste with the changing of CM/SA and C/M ratios. This is consistent with the regression analysis results of compressive strength shown as Equations (2) and (3), implying that among three chemical composition ratios S/A of slag shows the most significant impact on slag reactivity, hydration and strength development.

**Figure 19:**
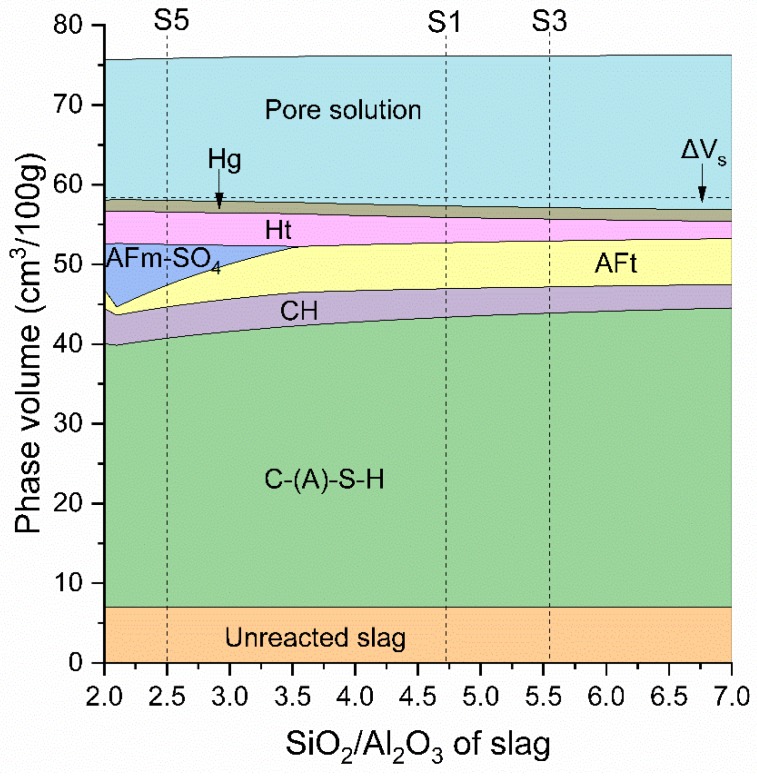
Modelled phase volume changes of slag blended paste with the effect of S/A ratio in slag.

To further explain the effect of S/A ratio, the modelled hydrated phases of slag blended paste changing with the S/A ratio of slag is shown in Figure 19. The model assumed the complete reaction of cement and 60% reaction of slag, which corresponds to the hydration situation after roughly 180 days or later. As observed in Figure 19, increasing the S/A ratio from that of S5 (S/A 2.49) to S1 (S/A 4.72), then to S3 (S/A 5.55) leads to the removal of AFm-SO_4_ phases (Ms and AFm_ss_) at S/A around 3.6, whilst increasing the volume of the C-(A)-S-H and AFt phases, slightly decreasing Ht phase and the total hydrate volume (seeΔV_s_ increases). The decrease in hydrate volume would result in the increase of porosity and thus reduce the compressive strength. This explains the difference in pore structure and strength results (Figure 6 and Figure 16) among blends containing S1, S3 and S5. Besides, it should be noted that this modelled phase volume changes (Figure 19) only reflect the influence of chemical compositions of slag, no kinetic effect of the three slags was considered. The difference in strength of slag blends was also attributed to the effect of hydration kinetics of slags as discussed in Table 7 and Figure 18.

The modelled phase changes in Figure 19 agree well with the phase evolution shown in XRD graph (Figure 9 and Figure 10). This reduction in C-(A)-S-H for slag blended cements was mainly due to the reduced SiO_2_ content in slag. Modelling also reveals that the type and amount of aluminate hydrates formed is greatly related to the S/A of slag. Lower S/A of slag promotes the formation of AFm-SO_4_ phases (Ms and AFm_ss_) which was experimentally observed using XRD as shown in Figure 10, when comparing CS5 with other two slag pastes. This is due to the increase of Al_2_O_3_ in slag, thus reducing the overall mass ratio of SO_3_/Al_2_O_3_ in the system and allowing more AFm-SO_4_ to precipitate at the expense of AFt [25]. Even though CS5 had less volume of C-(A)-S-H than CS1 and CS3 but had higher volume of aluminate phases. This suggests that the aluminate phases play an important role which might affect strength property. In addition, the modelled CH increases firstly and then starts to decrease slightly with the raising of S/A. The calculated CH evolution shows a slightly higher volume of CH for S5 than S1 and S3, which is different from the experimental result measured by TGA. This difference could be explained by two reasons. On one hand, the modelled phase volume is obtained by only changing the S/A of slag and keeping other oxides of slag constant, while in experiment besides S/A the ratios of C/M and CM/SA for slags S1,S3 and S5 were also varied. This would lead to the different CH volume between modelling and experiment. On the other hand, the faster hydration reaction of S5 than those of S1 and S3 would consume more CH and resulted in a lower amount of CH in the actual experiment.

## 4. Conclusions

In this study, the effect of different chemical compositions on the phase compositions of synthetic air quenched slag, the strength performance and hydration property of slag blends were investigated.

XRF results confirmed that the chemical compositions of synthetic air quenched slag were similar to the practical blast furnace slag. The main phases in synthetic air quenched slags were amorphous phase and some crystal phases such as gehlenite, akermanite, merwinite and bredigite. The amorphous content of air quenched slag, which depended on slag chemical compositions, varied from 33% to 100%. With the increase of the basicity CM/SA of slag, the amorphous content obviously decreased.

Synthetic air quenched slag mainly affected the performance of slag blends at later ages due to its slow reaction kinetics. The chemical compositions of air quenched slag showed more pronounced effect on compressive strength of slag blends than the amorphous content at 7 and 28 days. The S/A ratio of slag was found to be the dominant factor for the strength at 28 days and it showed a negative correlation with the compressive strength of slag blends, which was confirmed by the hydration investigation. The regression analysis also indicated the weak positive effect of CM/SA and C/M on strength at 28 days but there was no clear evidence to check this.

The hydration products of slag cement blends are C-(A)-S-H, CH, AFt with small amount of hydrotalcite, Ms and AFm_ss_. The contents of these hydrates are closely related to slag composition, especially S/A ratio. Thermodynamic modelling results have revealed that a higher S/A of slag leads to the increase of C-(A)-S-H and AFt contents, whilst decreasing the amounts of Ht and AFm-SO_4_ phases. The total volume of hydrates decreased slightly with the increase of S/A ratio of slag.

The results of experimental hydration investigation and thermodynamic modelling together confirmed that, the air quenching slag S5 with only 60.27 % amorphous content had higher DoR and solid phase volume than those of slags with almost 100% amorphous phase at 28 days. S/A of slag plays a major factor in the hydration of air quenched slag blends. A lower S/A could increase the reactivity and reaction degree of slag which were indicated by higher heat release, lower CH content, greater combined water amount and denser microstructure of hydrated paste, thus resulting in higher compressive strength.

## Figures and Tables

**Table 1:** Chemical compositions of industrial raw materials (wt.%).

Materials	Ignition Loss	SiO_2_	Fe_2_O_3_	Al_2_O_3_	CaO	MgO	SO_3_	K_2_O	Na_2_O
Limestone	42.14	3.75	0.38	0.78	51.11	1.33	0.09	0.33	-
Fly ash	2.01	48.15	3.75	37.01	4.2	0.6	0.47	1.16	0.35

**Table 2:** The designed chemical compositions of ten slags by uniform design method.

Synthetic Slag	CM/SA	C/M	S/A
S1	0.85	6.5	5.0
S2	0.90	11.5	3.0
S3	0.95	5.5	6.5
S4	1.00	10.5	4.5
S5	1.05	4.5	2.5
S6	1.10	9.5	6.0
S7	1.15	3.5	4.0
S8	1.20	8.5	2.0
S9	1.25	2.5	5.5
S10	1.30	7.5	3.5

**Table 3:** The proportions of raw materials for slag preparation (wt.%).

Synthetic Slag	Limestone	Fly Ash	MgO Reagent	SiO_2_ Reagent
S1	56.33	16.84	3.69	23.14
S2	59.39	24.35	1.79	14.47
S3	58.38	12.37	4.67	24.58
S4	62.51	16.24	2.18	19.07
S5	57.43	26.15	5.85	10.57
S6	64.82	11.67	2.61	20.90
S7	58.20	17.18	7.83	16.69
S8	64.26	27.15	2.98	5.61
S9	56.70	12.54	10.97	19.79
S10	66.60	16.78	3.65	12.97

**Table 4:** The Blaine specific surface area of air quenched slags and cement (m^2^/kg).

Cement	S1	S2	S3	S4	S5	S6	S7	S8	S9	S10
353	548	537	552	528	560	540	547	540	560	530

**Table 5:** Chemical compositions of air quenched slags determined by XRF (wt.%).

Oxides	S1	S2	S3	S4	S5	S6	S7	S8	S9	S10
SiO_2_	44.27	39.06	42.00	40.24	34.18	40.78	37.07	29.92	38.16	33.67
Al_2_O_3_	9.38	13.23	7.57	9.22	13.73	7.16	9.69	16.63	7.30	10.64
CaO	38.12	41.12	41.05	44.43	40.32	45.31	39.92	44.96	37.80	46.89
MgO	5.84	3.70	7.52	4.28	8.72	4.91	11.17	5.35	14.98	6.45
Fe_2_O_3_	1.13	1.43	0.95	1.06	1.47	0.91	1.09	1.54	0.88	1.14
SO_3_	0.08	0.08	0.05	0.10	0.09	0.05	0.06	0.08	0.06	0.04
K_2_O	0.49	0.59	0.45	0.52	0.60	0.48	0.50	0.65	0.42	0.54
Na_2_O	0.10	0.11	0	0	0.11	0	0	0.11	0	0.10
TiO_2_	0.37	0.44	0.26	0	0.52	0.26	0.34	0.53	0.25	0.35
MnO	0.03	0.02	0.02	0	0.02	0.02	0.02	0.03	0.02	0.02
P_2_O_5_	0.08	0.10	0.07	0.08	0.11	0.07	0.08	0.10	0.06	0.08
CM/SA	0.82	0.86	0.98	0.98	1.02	1.05	1.09	1.08	1.16	1.20
C/M	6.53	11.11	5.46	10.38	4.62	9.23	3.57	8.40	2.52	7.27
S/A	4.72	2.95	5.55	4.36	2.49	5.70	3.83	1.80	5.23	3.17

**Table 6:** Phase contents of air quenched slags determined by XRD-Rietveld analysis (wt.%).

Phases	S1	S2	S3	S4	S5	S6	S7	S8	S9	S10
Amorphous content	100.0	100.0	96.4	100.0	60.3	73.2	45.4	33.0	57.4	35.9
Gehlenite	-	-	-	-	10.6	-	-	32.9	-	20.9
Akermanite	-	-	3.6	-	13.1	-	8.7	1.9	-	11.4
Merwinite	-	-	-	-	16.0	-	45.9	32.2	42.6	31.8
Bredigite	-	-	-	-	-	26.8	-	-	-	-

**Table 7:** Modelled phase contents of slag blended pastes corresponding to the obtained 28-day DoR of slag (cm^3^/100g).

Paste	Phases in Slag Blended Paste								
	PC	Slag	C-(A)-S-H	CH	AFt	AFm-SO_4_	Ht	Hg	Pore Solution
CS1	1.59	12.90	24.66	4.80	3.58	0.00	1.95	1.31	27.80
CS3	1.59	11.80	25.75	5.00	3.58	0.00	2.05	1.32	27.01
CS5	1.59	10.98	25.11	4.41	0.73	4.91	3.21	1.60	25.03

## References

[1] Escalante-Garcia J.I., Espinoza-Perez L.J., Gorokhovsky A., Gomez-Zamorano L.Y. (2009). Coarse blast furnace slag as a cementitious material, comparative study as a partial replacement of Portland cement and as an alkali activated cement. Constr. Build. Mater..

[2] Taylor H.F.W. (1997). Cement Chemistry.

[3] Bellmann F., Stark J. (2009). Activation of blast furnace slag by a new method. Cem. Concr. Res..

[4] Kolani B., Buffo-Lacarrière L., Sellier A., Escadeillas G., Boutillon L., Linger L. (2012). Hydration of slag-blended cements. Cem. Concr. Compos..

[5] Ramezanianpour A.A. (2014). Cement Replacement Materials—Properties, Durability, Sustainability.

[6] Kumar R., Kumar S., Badjena S., Mehrotra S.P. (2005). Hydration of mechanically activated granulated blast furnace slag. Metall. Mater. Trans. B Process Metall. Mater. Process. Sci..

[7] Kumar S., Kumar R., Bandopadhyay A., Alex T.C., Kumar B.R., Das S.K., Mehrotra S.P. (2008). Mechanical activation of granulated blast furnace slag and its effect on the properties and structure of portland slag cement. Cem. Concr. Compos..

[8] Haha M.B., Saout G.L., Winnefeld F., Lothenbach B. (2011). Influence of activator type on hydration kinetics, hydrate assemblage and microstructural development of alkali activated blast-furnace slags. Cem. Concr. Res..

[9] Li C., Sun H., Li L. (2010). A review: The comparison between alkali-activated slag (Si+Ca) and metakaolin (Si+Al) cements. Cem. Concr. Res..

[10] Shi C., Jiménez A.F., Palomo A. (2011). New cements for the 21st century: The pursuit of an alternative to Portland cement. Cem. Concr. Res..

[11] Juenger M.C., Winnefeld F., Provis J.L., Ideker J.H. (2011). Advances in alternative cementitious binders. Cem. Concr. Res..

[12] Haha M.B., Lothenbach B., Saout G.L., Winnefeld F. (2011). Influence of slag chemistry on the hydration of alkali-activated blast-furnace slag—Part I: Effect of MgO. Cem. Concr. Compos. Concr. Res..

[13] Maruoka N., Mizuochi T., Purwanto H., Akiyama T. (2004). Feasibility Study for Recovering Waste Heat in the Steelmaking Industry Using a Chemical Recuperator. ISIJ Int..

[14] Barati M., Esfahani S., Utigard T.A. (2011). Energy recovery from high temperature slags. Energy.

[15] Zhang H., Wang H., Zhu X., Qiu Y.-J., Li K., Chen R., Liao Q. (2013). A review of waste heat recovery technologies towards molten slag in steel industry. Appl. Energy.

[16] Purwanto H., Mizuochi T., Tobo H., Takagi M., Akiyama T. (2005). Characteristics of Glass Beads from Molten Slag Produced by Rotary Cup Atomizer. Mater. Trans..

[17] Mizuochi T., Akiyama T., Shimada T., Kasai E., Yagi J. (2001). Feasibility of Rotary Cup Atomizer for Slag Granulation. ISIJ Int..

[18] Bisio G. (1997). Energy recovery from molten slag and exploitation of the recovered energy. Energy.

[19] Liu J., Yu Q., Zuo Z., Yang F., Duan W., Qin Q. (2017). Blast furnace slag obtained from dry granulation method as a component in slag cement. Constr. Build. Mater..

[20] Liu J., Yu Q., Zuo Z., Yang F., Han Z., Qin Q. (2019). Reactivity and performance of dry granulation blast furnace slag cement. Cem. Concr. Compos..

[21] Pal S.C., Mukherjee A., Pathak S.R. (2003). Investigation of hydraulic activity of ground granulated blast furnace slag in concrete. Cem. Concr. Res..

[22] Lothenbach B., Scrivener K., Hooton R.D. (2011). Supplementary cementitious materials. Cem. Concr. Res..

[23] Gu K., Jin F., Al-Tabbaa A., Shi B., Liu J. (2014). Mechanical and hydration properties of ground granulated blastfurnace slag pastes activated with MgO-CaO mixtures. Constr. Build. Mater..

[24] Mostafa N.Y., El-Hemaly S.A.S., Al-Wakeel E.I., El-Korashy S.A., Brown P.W. (2001). Characterization and evaluation of the hydraulic activity of water-cooled slag and air-cooled slag. Cem. Concr. Res..

[25] Whittaker M., Zajac M., Ben Haha M., Bullerjahn F., Black L. (2014). The role of the alumina content of slag, plus the presence of additional sulfate on the hydration and microstructure of Portland cement-slag blends. Cem. Concr. Res..

[26] Wang P.Z., Trettin R., Rudert V., Spaniol T. (2004). Influence of Al_2_O_3_ content on hydraulic reactivity of granulated blast-furnace slag, and the interaction between Al_2_ O_3_ and CaO. Adv. Cem. Res..

[27] Fang K.T., Ma C., Winker P., Zhang Y. (2000). Uniform Design: Theory and Application. Technometrics.

[28] Li Z., Du S. (2010). Optimization of Experimental Design and Statistical Analysis.

[29] GB/T 17671-1999, Method of Testing Cements-Determination of Strength (ISO). http://www.lancarver.com/UpFiles/pdf/2014-04-08/040809453417.pdf.

[30] Haha M.B., Lothenbach B., Le Saout G., Winnefeld F. (2012). Influence of slag chemistry on the hydration of alkali-activated blast-furnace slag—Part II: Effect of Al_2_O_3_. Cem. Concr. Res..

[31] Kulik D.A. GEM-Selektor v.3. http://gems.web.psi.ch/.

[32] Wagner T., Kulik D.A., Hingerl F.F., Dmytrieva S.V. (2012). GEM-Selektor Geochemical Modeling Package: TSolMod Library and Data Interface for Multicomponent Phase Models. Can. Mineral..

[33] Kulik D.A., Wagner T., Dmytrieva S.V., Kosakowski G., Hingerl F.F., Chudnenko K.V., Berner U.R. (2013). GEM-Selektor geochemical modeling package: Revised algorithm and GEMS3K;numerical kernel for coupled simulation codes. Comput. Geosci..

[34] GEMS Default Thermodynamic Database. http://gems.web.psi.ch/TDB/index.html.

[35] Thermodynamic Data. https://www.empa.ch/web/s308/thermodynamic-data.

[36] Lothenbach B., Kulik D.A., Matschei T., Balonis M., Baquerizo L., Dilnesa B., Miron G.D., Myers R.J. (2019). Cemdata18: A chemical thermodynamic database for hydrated Portland cements and alkali-activated materials. Cem. Concr. Res..

[37] Snellings R., Paulhiac T., Scrivener K. (2014). The Effect of Mg on Slag Reactivity in Blended Cements. Waste Biomass Valoriz..

[38] Hesse C., Goetz-Neunhoeffer F., Neubauer J. (2011). A new approach in quantitative in-situ XRD of cement pastes: Correlation of heat flow curves with early hydration reactions. Cem. Concr. Res..

[39] Jansen D., Goetz-Neunhoeffer F., Stabler C., Neubauer J. (2011). A remastered external standard method applied to the quantification of early OPC hydration. Cem. Concr. Res..

[40] Jansen D., Goetz-Neunhoeffer F., Lothenbach B., Neubauer J. (2012). The early hydration of Ordinary Portland Cement (OPC): An approach comparing measured heat flow with calculated heat flow from QXRD. Cem. Concr. Res..

[41] Schöler A., Lothenbach B., Winnefeld F., Zajac M. (2015). Hydration of quaternary Portland cement blends containing blast-furnace slag, siliceous fly ash and limestone powder. Cem. Concr. Compos..

[42] Deschner F., Winnefeld F., Lothenbach B., Seufert S., Schwesig P., Dittrich S., Goetz-Neunhoeffer F., Neubauer J. (2012). Hydration of Portland cement with high replacement by siliceous fly ash. Cem. Concr. Res..

[43] Deschner F., Lothenbach B., Winnefeld F., Neubauer J. (2013). Effect of temperature on the hydration of Portland cement blended with siliceous fly ash. Cem. Concr. Res..

[44] Taylor R., Richardson I.G., Brydson R. (2010). Composition and microstructure of 20-year-old ordinary Portland cement—Ground granulated blast-furnace slag blends containing 0 to 100% slag. Cem. Concr. Res..

[45] Kumar S., Bandopadhyay A., Rajinikanth V., Alex T.C., Kumar R. (2004). Improved processing of blended slag cement through mechanical activation. J. Mater. Sci..

